# Explore Interregional EEG Correlations Changed by Sport Training Using Feature Selection

**DOI:** 10.1155/2016/6184823

**Published:** 2016-01-10

**Authors:** Jia Gao, Wei Wang, Ji Zhang

**Affiliations:** ^1^Laboratory of Machine Learning and Cognition, Nanjing Normal University, Nanjing 210097, China; ^2^Faculty of Health, Engineering and Sciences, University of Southern Queensland, Toowoomba, QLD 4350, Australia

## Abstract

This paper investigated the interregional correlation changed by sport training through electroencephalography (EEG) signals using the techniques of classification and feature selection. The EEG data are obtained from students with long-time professional sport training and normal students without sport training as baseline. Every channel of the 19-channel EEG signals is considered as a node in the brain network and Pearson Correlation Coefficients are calculated between every two nodes as the new features of EEG signals. Then, the Partial Least Square (PLS) is used to select the top 10 most varied features and Pearson Correlation Coefficients of selected features are compared to show the difference of two groups. Result shows that the classification accuracy of two groups is improved from 88.13% by the method using measurement of EEG overall energy to 97.19% by the method using EEG correlation measurement. Furthermore, the features selected reveal that the most important interregional EEG correlation changed by training is the correlation between left inferior frontal and left middle temporal with a decreased value.

## 1. Introduction

Motor skill learning refers to the process by which movements are executed more quickly and accurately with sport training [1]. Acquisition of motor skills is paralleled by changes at the regional level and the level of neural circuitry. The brains of sport experts could be regarded as the ideal subjects to explore the relationship between cerebral plasticity and learning of complex motor skill [2, 3]. In recent years, data from a large number of functional imaging studies, as well as electroencephalography (EEG) studies, have not only provided insight into the neuronal mechanisms underlying changes that occur during motor skill acquisition but also achieved a better understanding of plastic changes that occur within these neural systems as performance improves with practice of a motor task [4–6].

There has been increasing interest in applying connectivity analysis to brain measures. The relation between resting-state functional connectivity of brain areas activated during a divergent thinking task and the effect of practice in 32 adolescents was tested [7]. Differences in the interaction of large-scale brain networks are examined when subjects are learning with different training strategies [8]. The association between aerobic fitness, functional connectivity in the default mode network (DMN), and cognitive performance was also examined [9]. Most of these studies have relied on fMRI, which substantially limits the participant groups and numbers that can be studied. Electroencephalography (EEG) has been widely used to investigate the human brain since Hans Berger made the first human EEG recording in the middle of the 1920s [10]. EEG recordings offer a comparatively inexpensive and easy-to-use alternative. The advantages offered by EEG-based approaches are their spatiotemporal resolution and the potential to preserve ecological validity, that is, to obtain measurements of cortical function under the same conditions that the task is normally performed. EEG signals can change under special mental tasks and reflect different developmental patterns in brain after long-term different professional training [11].

Resting-state studies have been used for measuring differences in brain function in relation to behavior across individual subjects [12]. Resting state cortical connectivity reflected in EEG coherence in individuals with autism was investigated [13]. Coherence in resting EEG data was a strong predictor of motor skill acquisition [14]. Task-specific modulation of EEG connectivity during two simple unimanual motor tasks was reported by Herz et al. [15]. These studies have focused on resting state EEG coherence or task-specific EEG connectivity. However, few studies have examined EEG coherence under imagery tasks and the effects of general physical training on neural networks. Also, most of the studies only investigate the motor network but not the whole brain network. As a complex dynamic system, the structure and function of brain will repair and recombine dynamically under the influence of learning, training, experience, and other factors.

The aim of this study is to explore the EEG correlation patterns changed by sport training. We assume that the whole brain EEG network under imagery tasks provides some insightful findings in the effect of sport training. In this paper, we took EEG signals acquired from two groups of participants when they were performing motor imagery tasks. Discrete Wavelet Transform (DWT) is used to extract frequency features which are used to construct second order measurements, correlation features. And then Partial Least Square (PLS) analysis is utilized for feature selection. PLS modeling is used for defining relationships between brain function and behavior for the motor imagery tasks. Based on this, we investigate the significant EEG correlations affected by sport training.

It is worthwhile highlighting the advantages of our study. Firstly, the use of EEG may offer some advantages for measuring synchronized activity in cross-neural ensembles [14] as EEG provides higher temporal resolution than many other functional neuroimaging techniques. Such high temporal resolution may have been particularly advantageous in the current study as it permits measurement of brain function in different frequencies. We used Discrete Wavelet Transform (DWT) to extract frequency features. Secondly, we are interested in the training-induced EEG correlations in this study. Connectivity-based measures, as compared to focal measures of brain activity, have an improved ability to provide insight into cortical processing underlying complex behaviors [16]. Pearson correlation is utilized to calculate EEG correlation. Thirdly, the metric of motor imagery provides an easier and more targeted way to study training-induced brain changes. In the present study, EEG data were acquired when training group and baseline group performed motor imagery tasks.

The key scientific contributions of this paper are summarized as follows:

- The use of measurement of EEG correlations provides potential biomarkers associated with sport training and hence is of great interest to researchers. Specifically, every lead is considered as a node of the brain network and the interregional correlations based on EEG are extracted, including correlation of EEG overall energy and correlations of different frequency bands. EEG overall energy is EEG signals before decomposing. Based on the result of Support Vector Machine (SVM) classification, measurement of EEG correlations is more effective to verify different training levels.
- We utilize Partial Least Square (PLS) analysis for feature selection in our method which is a supervised learning method. PLS modeling is very useful for defining relationships between brain function and behavior [17]. This kind of feature selection makes our analysis much easier by avoiding the huge calculation. EEG overall energy correlation and beta correlation are also compared to investigate variation between subjects in different training levels.

## 2. Method

In this section, we describe motor imagery tasks that the subjects performed for the acquisition of EEG data. Data preprocessing and feature extraction of EEG signals are presented. In our study, Support Vector Machine (SVM) is used to perform classification and Partial Least Square is utilized for feature selection.

### 2.1. Motor Imagery Task

Findings from previous studies of motor imagery have suggested that the performance of a motor task and its imagination share common neural substrates [18]. Imagining the motor tasks from a first person perspective is called motor imagery (MI), which is defined as a dynamic state during which a subject simulated an action mentally without any overt body movement [19]. Lafleur et al. [20] have demonstrated that the cerebral plasticity that occurs following physical practice is reflected during MI. The effects of training with a MI-based brain-computer interface on activation patterns of the sensorimotor cortex are studied [21]. Comparisons between expert athletes and novices demonstrated different patterns of brain activation during motor imagery of the corresponding task [22]. Therefore, motor imagery tasks can be used to test the effects of motor practice effectively.

Reasonable mental tasks can induce clear and strong EEG signals. There are two different experimental conditions in this work, that is, a rest condition and an active one. In the rest condition, participants need to be relaxing with eyes closed. In the active condition, there are two mental tasks, high jumping imagery and swimming imagery, during both of which participants imagine from the first person perspective. Participants watch a video of imagery content before the experiment to guarantee that they imagine the same thing. The two mental tasks constitute a continuous stimulus, which can lead to repeated activation of the brain regions associated with motor imagery and induce EEG changes.

The experiment is designed to make the rest and the active conditions alternate with each other every 60 seconds in a repeated manner. More specifically, the motor imagery tasks of this experiment are designed as follows: (1) close eyes and relax without any imagination for one minute; (2) imagine doing high jump (Task (1)) for one minute with eyes closed; (3) close eyes and relax without any imagination for one minute; and (4) imagine doing swimming (Task (2)) for one minute with eyes closed. Participants had to perform such motor imagery repeatedly until they heard the voice saying “stop.” Eight subjects completed the aforementioned tasks in sequence under the guidance of the experiment operator.

### 2.2. EEG Acquisition and Preprocessing

The EEG data used in our work were generated from the Machine Learning and Cognition Laboratory of Nanjing Normal University, China. College students from the university were chosen as the research objects in our study. In this work, two groups of students were created and studied: the sport training group consists of students who practice full-time motor skill over one year, while the non-sport training group consists of healthy participants who are not involved in any extensive sport training. EEG data were collected from eight college students who were selected from the Physical Education Department and other departments. There were equal number of males and females in each group. The age of all participants ranged from 20 to 24 years. The selected subjects were all in good health without mental disorders and organic diseases. The basic cognitive ability test (version 2.0) and the Raven test screening demonstrated that they all have normal intelligence and cognitive ability. Participants were right-handed as revealed by self-report. After introducing the detailed experimental purposes and processes, the subjects were voluntary to participate in the experiments and actively cooperated with the experimental operator to complete the experiment.

The data collection equipment is the latest EEG acquisition system developed by Nanjing WEISI (VISH) company. Experimental EEG data were recorded by Vishcap-19-lead electrode cap, adopting the international 10/20 lead standard. EEG signals were digitized at a sample rate of 512 Hz. Following the experimental steps, we got eight EEG series under Task (1) and eight EEG series under Task (2) from eight subjects. Each EEG series is one minute long.

The obvious eye movement, body movement, and interference signals caused by noise were manually removed first, as suggested by the experimental reports and related events records [11]. In addition, subjects typically need a few seconds to fall into effective thinking and the last few seconds are often not very effective, so the EEG data are generally taken in the middle time period when the subjects feature better mental state and concentration. In this study, we take 40 s EEG data in the middle from each one-minute EEG series.

After the above two steps, the EEG data passed the 0.5–40 Hz digital bandpass filter. We divide each 40 s EEG series into 20 samples to expand the sample size. The length of each sample (time series) is set as 1024 (2 s) due to the following reason. EEG data covering sampling frequency are more representative and the experimental sampling frequency is chosen as 512 Hz. In the end, we obtained 160 samples (20 samples × 2 tasks × 4 subjects) of training group and 160 samples (20 samples × 2 tasks × 4 subjects) of non-training group.

### 2.3. Feature Extraction Based on Functional Network

We utilize the Discrete Wavelet Transform (DWT) to analyze signals at different frequency bands in different resolutions by decomposing them into two parts, including a coarse approximation and detailed information which are explained later in Figure 2. DWT employs two sets of functions called* scaling functions* and* wavelet functions*, which are related to low-pass and high-pass filters, respectively. Decomposition of signals into different frequency bands is merely obtained by consecutive high-pass and low-pass filtering of the time domain signal [23].

**Figure 1:**
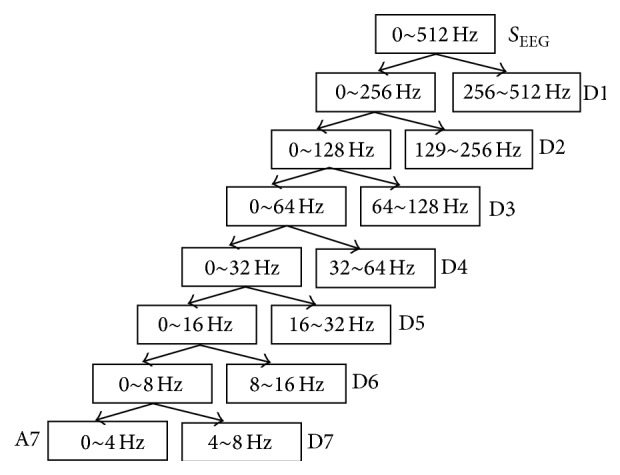
Subband decomposition of discrete wavelet transform (DWT) implementation.

Selection of suitable wavelet and the number of decomposition levels are very important in the analysis of signals using the DWT. Usually, tests are performed with different types of wavelets and the one which gives maximum efficiency is selected for the particular application [23]. The smoothing feature of the Daubechies wavelet of order 4 (db4) makes it more appropriate to detect changes of EEG signals than other wavelets which are Symmlet of order 10 (sym10), Coiflet of order 4 (coif4), and Daubechies of order 2 (db2) [23]. The wavelet coefficients are computed using the db4 in our study and this method was applied on the EEG data. The number of decomposition levels is chosen based on the dominant frequency components of the signals. The levels are chosen such that those parts of the signal that correlate well with the frequencies and are necessary for signal classification are retained in the wavelet coefficients. The convention is to obtain four frequency components from EEG recordings, which are delta (*δ*) (±0 to 4 Hz), theta (*θ*) (4–8 Hz), alpha (*α*) (8–13 Hz), and beta (*β*) (13–20 Hz) [24]. The number of decomposition levels is chosen to be seven in our study, so that we can get the useful four frequency bands. As a result, the EEG signals are decomposed into detailed components D1–D7 and one final approximation, A7.  Figure 1 shows approximation (A7) and detailed component (D1–D7) of an EEG signal. We can see from Figure 1 that A7, D7, D6, and D5 are, respectively, corresponding delta, theta, alpha, and beta bands. By this step, we get four frequency bands of 320 samples which are the basis to construct EEG correlation feature.

After decomposition of signals is completed, brain networks can be constructed for each subject, from which informative features are extracted for classification. Take the brain network of overall energy, for example; its nodes correspond to the energy of a specific EEG lead, and its undirected edges correspond to the interactions between energy of two nodes. In our study, the number of nodes is 19, which is the number of EEG lead. The edge values, the Pearson correlations between every two connected nodes, are concatenated to form the feature vectors for use in classification.

The Pearson Correlation Coefficient (PCC) is a well-established measure of correlation. It was introduced by Galton [25] and developed later by Pearson [26]. Pearson Correlation Coefficient is related to the covariance and defined as

(1) $$\begin{array}{c} R\left(\;i,j\;\right)=\frac{C\left(i,j\right)}{\sqrt{C\left(i,i\right)C\left(j,j\right)}}. \end{array}$$

**R**  is the Pearson Correlation Coefficient matrix which is a symmetric 19 × 19 matrix for each EEG sample, and **C** is the covariance matrix. PCC measures the linear relationship between two random variables and has a range from +1 (perfect correlation) to −1 (perfect but negative correlation), with 0 denoting the absence of a relationship. In our approach, we keep all the connections so that new relationships between energy changes and the training are not left unexplored. We directly use the Pearson correlation in the brain network as features; that is, we concatenate the elements in the upper triangle matrices of correlation matrices computed above due to its symmetry. We obtain 171 features for each of the 320 samples after concatenating. This method is applied to EEG overall energy as well as four frequency bands and then we get correlation features.

Support Vector Machine (SVM) is considered as an effective technique for statistical estimation and prediction of learning on a small number of samples; thus, the SVM classifier is chosen in our work for classifying EEG signals. In this work, experimental samples were classified by applying* svmtrain* and* svmclassify* in function library of Matlab 2010b. The input parameters for* svmtrain* are EEG samples and corresponding labels and kernel function. There are three kinds of EEG samples which are EEG overall energy features, EEG overall energy correlation, and four frequency bands correlations. Samples from training group are labeled “1” and those from the other group “0”. We choose* polynomial* as the kernel function after comparing the classification results of different kernel functions. We compare the discrimination power of EEG overall energy correlation and EEG energy features. Our network features differ from the conventional energy features in that the network features model the regional interactions. Therefore, two kinds of features are, respectively, used to train a linear SVM for predicting sport training group and baseline group. We also compare the efficacy of EEG overall energy correlation and four frequency bands correlation with respect to classification. The training and testing samples are selected randomly by the program. The data set is randomly partitioned into 10 training and test groups with 288 samples for training and 32 samples for test. The output of classification is the accuracy in predicting sport training group and baseline group. The results were obtained by 10-fold cross-validation.

### 2.4. Feature Selection Based on PLS

To identify the discriminative EEG correlation features for classification, Partial Least Square (PLS) is utilized to sort the feature vector in terms of contribution to classification. As a supervised learning method, PLS takes into account the information in the classification labels and thus achieves a better discrimination than many of the commonly used unsupervised methods [27] such as Principal Components Analysis (PCA) and the Laplacian Eigenmap. PLS models the relations between the predictive variables (the feature vector **X**) and the target variables (the label vector **Y**) by means of latent variables. PLS maximizes the covariance of the projections of **X** and **Y** to latent structures, as well as the individual variance of **X** and **Y**. This method is particularly suitable for the datasets where the size of samples is much smaller than the size of features.

Specifically, let the *n* × *d* matrix **X** represent the *d*-dimensional feature vectors with *n* subjects, and let **Y** represent the corresponding 1-dimensional label vector. PLS decomposes the zero-mean matrix **X** and vector **Y** into

(2) X=TPT+E,Y=UQT+F,

where **T** and **U** are *n* × *p* matrices containing *p* extracted latent vectors, the *d* × *p* matrix **P** and the 1 × *p* vector **Q** represent the loadings, and the *n* × *d* matrix **E** and the *n* × 1 vector **F** are the residuals. The latent matrices **T** and **U** have the following properties: each column, called a latent vector, is a linear combination of the original variables **X** and **Y**, respectively, and the covariance of two latent vectors **t**_*i*_ and **u**_*i*_ is maximized.

The PLS model was trained by a function named* simpls* in Matlab 2010b. We obtained **T**, **U**, and **w** from the function* simpls.* **T** and **U** are *n* × *p* matrices containing *p* extracted latent vectors, and **w** is the optimal weight vector. After that, a method called Variable Importance on Projection (VIP) [28] is used to rank these features according to their discriminative power in the learned PLS model. The discriminative power is measured by a VIP score. The higher the VIP score is, the more discriminative the feature is. A VIP score for the *j*th feature is defined as

(3) $$\begin{array}{c} {VIP}_{j}=\sqrt{\frac{d\sum_{k=1}^{p}\rho_{k}^{2}w_{jk}^{2}}{\sum_{k=1}^{p}\rho_{k}^{2}}}, \end{array}$$

where *d* is the number of features, *p* is the number of the latent vectors as defined above, *w*_*jk*_ is the *j*th element in the vector **w**_*k*_, and *ρ*_*k*_ is the regression weight for the *k*th latent variable; that is, *ρ*_*k*_ = **u**_*k*_^*T*^ **t**_*k*_.

## 3. Results

In our work, we conduct two comparative studies. We first compare the performance of EEG overall energy correlation and EEG overall energy features and then compare the efficacy of EEG overall energy correlation and four frequency bands correlation. Note that, as the number of correlation features is large, feature selection is applied to obtain the most important and discriminative features. Furthermore, we present the value of those selected features in order to facilitate the investigation of specific variation of EEG correlations.

### 3.1. Comparison of Different EEG Measurements

The classification results using different measurements are shown in Table 1. The classification accuracy in Table 1 is the result averaged by 10-fold cross-validation. Without requiring new sources of information, using EEG overall energy correlation improves the accuracy of predicting subjects with different training level from 88.13% to 97.19% compared with using EEG energy measurement directly. The advantage may come from using regional interactions. Furthermore, we perform classification using four frequency bands correlation. The classification result is shown in Table 1. Filtering out all but the beta band gives performance close to using the full signal, suggesting that most of the information is in the beta band.

### 3.2. Feature Selection Related to Motor Imagery Task

The conventional method using EEG energy directly [4, 29] exploits the absolute changes of EEG among different subjects. In this work, we study the interregional correlations of EEG which are the relative changes of EEG in different leads of the same subject. Using interregional correlations of EEG can help eliminate the impact of personal variations in motor imagery task. To do this, we first apply feature selection based on EEG overall energy correlation because this measurement produces the highest classification accuracy. As the classification accuracy with beta correlation as the measurement is obviously higher than others and is close to that of EEG overall energy correlation, we also apply feature selection on beta correlation.

To find out the optimal minimum number of features to be selected, we conduct a comparison of classification accuracy using EEG overall energy correlations with different feature numbers. These features are ranked by their VIP scores in PLS-based feature selection. The higher the score is, the more discriminative the feature is. Each classification is conducted ten times using certain number features and the mean classification accuracy is shown in Table 2. We can see from the table that the values are all similar and it is the largest using ten features; thus the number of features is set to ten in the following study.

**Figure 2:**
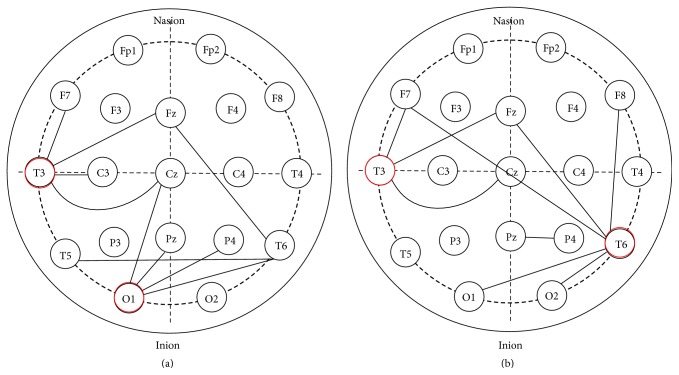
Distribution of selected features from EEG overall energy correlation (a) and beta correlation (b).

We found that classification accuracy got to 92.69% using the first ten EEG overall energy correlation features and 89.50% using the first ten beta correlation features, indicating that these ten interregional EEG correlations are really important. A *t*-test is conducted with each selected feature to demonstrate the efficacy of our method.  Table 3 and Figure 2 show the most discriminative features selected in our experiment based on their VIP scores and the corresponding *p* value. The most discriminative EEG energy correlation feature is Feature #137. All of the ten features in energy correlation are statistically significant (*p* < 0.05). According to the correlation matrix, Feature #137 is the correlation between 11 and 13 in original energy features, corresponding to left inferior frontal (F7) and left middle temporal (T3), respectively. We can see from Figure 2(a) that among these ten correlations, four regions are related to left middle temporal (T3), and the other four regions are all related to left occipital (O1).

As to the beta correlation, the first feature selected is Feature #167, which is the sixth in EEG overall energy correlation. Most of the ten features in beta correlation are statistically significant (*p* < 0.05) except for Feature #114, so Feature #114 is no longer considered afterwards. Feature #167 corresponds to T6-Fz according to the correlation matrix.  Figure 2(b) shows that five regions are related to T6 in all, and three regions interacted with T3. For both measurements, Feature #123 is ranked second.

### 3.3. Comparative Study on EEG Correlation

**Figure 3:**
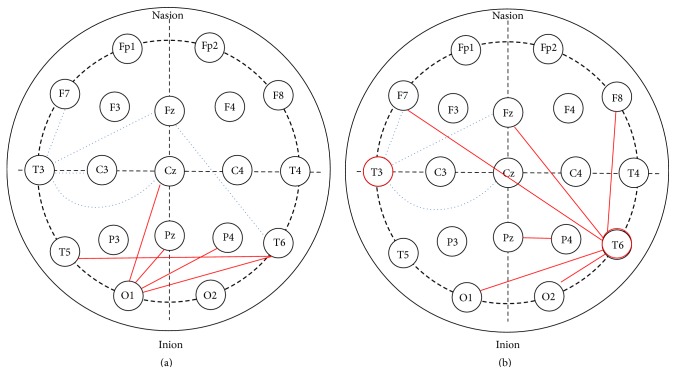
Variations of interregional EEG correlations. (a) is in the measurement of EEG overall energy correlation. (b) is in the measurement of beta correlation. Blue dotted lines indicate the decreased correlation coefficient affected by sport training. Red solid lines indicate the increased correlation coefficient affected by sport training.

To identify the difference of EEG correlation between the training group and baseline group, a comparative study is carried out which uses the mean value of the features. As there are both positively and negatively correlated features, the mean value was calculated by the absolute value of all correlation in each group.  Table 4 (left) shows the result of EEG overall energy correlation in training group and baseline group selected in Section 3.2. The first EEG correlation between left inferior frontal and left middle temporal in training group (0.45) is lower than baseline group (0.67), while the second distinct EEG correlation between left occipital and right posterior temporal in training group (0.54) is higher than baseline group (0.37). It shows that EEG correlation between left inferior frontal and left middle temporal is weaker while that between left occipital and right posterior temporal is stronger after long term training.  Figure 3(a) shows the change of interregional EEG overall energy correlation affected by sport training. All the correlations related to left middle temporal (T3) are lower in training group, and all correlations related to left occipital (O1) are higher in training group compared to baseline group. In addition, right middle temporal-middle frontal (Fz-T6) is a feature with a decreased value and left posterior temporal-right posterior temporal (T5-T6) is a feature with an increased value affected by sport training.

Table 4 (right) shows the result of beta correlation in training group and baseline group. The first four discriminative features in training group were all higher than baseline group. It can be seen from Figure 3(b) that all the correlations related to left middle temporal (T3) are lower in training group, which is consistent with EEG overall energy correlation. Also, all the correlations related to right posterior temporal (T6) are higher in training group than those in baseline group.

**Figure 4:**
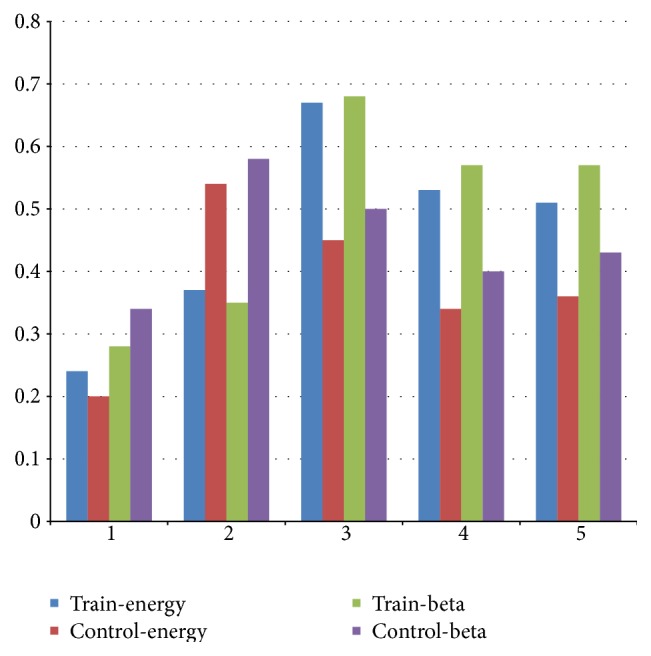
Mean correlation of five common features. 1–5 represent Features T6-Fz, O1-T6, F7-T3, T3-Fz, and T3-Cz.

Five EEG correlations are further investigated for both measurements of EEG overall energy correlation and beta correlation, which are T6-Fz, O1-T6, F7-T3, T3-Fz, and T3-Cz. The mean values of measurements for these features are shown in Figure 4. Most of the variations are consistent for all features between training group and baseline group except T6-Fz. The value of correlation for O1-T6 is lower in training group than nontraining group for both measurements, and the value of correlation for F7-T3, T3-Fz, and T3-Cz is higher in training group than nontraining group for both measurements at the same time.

## 4. Discussion

Analysis of electroencephalography (EEG) energy is a useful technique in the brain signal processing. EEG frequency bands may best be used to develop an EEG-based brain-computer interface (BCI) for the decoding of 3D hand motion trajectories [29]. Spectral modulation of frontal EEG during motor skill acquisition was investigated and significant decrease of delta, theta, and gamma activities was found after repeated practice [4]. Studies on fMRI and MEG have confirmed that neural correlates are associated with motor skills. Professionals reveal experience-related neural networks of the brain, which might account for their excellent motor performance [2]. Our study examines the ability of EEG correlation to predict the influence of general sport training on individuals. We found that there exists more obvious difference in interregional EEG correlation than EEG energy between training group and baseline group when the subjects are performing motor imagery tasks. Similar studies have been carried out earlier. Herz et al. [15] revealed dominant coupling within the *β*-band (13–30 Hz) between M1 and SMA during isometric contraction of the forearm. A stronger influence of motor beta on occipital gamma was found for slow responses [30]. We demonstrated with this study that interregional EEG correlation is a more accurate measure for demonstrating the effect of general sport training than EEG energy.

Our approach not only predicts training effect but also provides insight into the EEG correlations underlying this training effect. Our results demonstrate that correlation between left inferior frontal and left middle temporal (F7-T3) is most discriminative among the subjects with a lower value in training group. This might be interpreted as better brain efficiency in motor imagery for training group. Sami and Miall [31] found a change in local efficiency in the inferior frontal and cerebellar regions distinguishes between procedural learning and the joystick learning. Farah et al. [32] in their event-related potentials study found an effect of imagery within 200 ms localized at the occipital and posterior temporal regions. Temporal lobe is mainly in charge of identification of hearing, speech comprehension, and memory. Frontal lobe is responsible for language, emotion, and some auxiliary motor function, affecting planning and organizing of movement [33]. The decreased correlation between left inferior frontal and left middle temporal suggests that auxiliary motor function is less associated with speech comprehension in training group. This might be explained by behavior automation after long-term sport training. In this study, when the training group receives motor imagery tasks, they can perform corresponding activities automatically without complex speech comprehension.

Generally speaking, these significant EEG overall energy features are focused on left middle temporal (T3) and left occipital (O1) which closely resemble the hubs described in the fMRI literature [34]. Features related to the former are suppressed while features associated with the latter are strengthened in the training group. Chang et al. [35] compared the activation maps of elite archers and nonarchers during mental rehearsal of archery and found that the neural correlates of elite archers were more focused and efficiently organized than those of nonarchers. Strengthened correlations with left occipital are in accordance with this study which suggests that left occipital plays a more important role in the training group. This area becomes closer to other areas affected by sport training. However, it is possible that suppressed correlations with left middle temporal happen by the measurement of EEG overall energy correlation. We consider that the relations of left middle temporal with other areas are weakened after sport training because the training group rely more on their muscle memory other than hearing or speech comprehension. This provides new insight into the effect of general sport training. Farah et al. [36] reported a laterality of an involvement of visual imagery, greater at the left posterior temporal side than the right. There is also a laterality at the left side in motor imagery tasks. We get a similar result in this study that the two hubs are both at the left side. The remaining two EEG correlations, left posterior temporal-right posterior temporal (T5-T6) and right posterior temporal-middle frontal (T6-Fz), are difficult to explain in the study, which will involve further investigations.

The excellent temporal resolution of the EEG allows the different physiological processes to manifest in different frequency bands. In this study, motor imagery tasks are found to be associated with predominant interregional coupling in the *β*-band. Analysis of beta correlation shows a consistent variation with EEG overall energy correlation between training group and baseline group. This is in accordance with the results from previous electrophysiological studies [37]. Beta activity is closely associated with function of the cortical motor system [38]. In our study, beta band is shown to be the best biological correlate and the prediction strength was weaker in alpha, delta, and theta frequency ranges. Coupling in the *β*-band during tonic movements has been observed between cortical regions as well as in corticomuscular connections [39] and probably plays an important role in preserving the motor-state [40]. Analyzing beta correlation yields further informative results. Strong beta correlations with right posterior temporal (T6) are expressed in the sport training group, indicating that beta band of right posterior temporal plays a vital role in the interaction of brain regions after training.

## 5. Conclusion

In this paper, we investigate how the effect of sport training might be manifested in different college majors and whether interregional correlation of EEG allows additional insights to changes in neural activity with continued sport training. Using a brain network, we show that the training group and baseline group are characterized by very distinct patterns. These differences are best captured by a model postulating training-induced changes in interregional EEG correlation. Based on this model, we are able to identify the specific variation of EEG correlation. Training group reveals some decreased correlations with left middle temporal and some increased correlations with left occipital. Changes in these functional areas can provide the basis for more effective sport training. The result can also lay the foundation for more accurate and objective evaluation of human's athletic ability.

Our current study is subject to some limitations that we will try to overcome in our future research work. We acknowledge that the use of scalp EEG is limited in spatial localization and the number of participants can be further increased. The anatomical relationship between EEG electrodes and specific brain structures is imperfect using traditional 10-20 systems. Inconsistent change of beta correlation T6-Fz with EEG overall energy correlation is left to be explored. In the future, we could acquire more high density EEG recordings to overcome the limitations. Our future work on the evolution of connectivity profiles in parallel with behavioral gains might provide additional insights into the mechanisms underlying motor learning.

## Figures and Tables

**Table 1:** Classification accuracy of different measurements.

Measurement	Classification accuracy (%)
MI_energy	88.13 ± 4.81
MI_energy_cor	97.19 ± 1.75
MI_alpha_cor	79.00 ± 4.62
MI_beta_cor	91.44 ± 3.81
MI_delta_cor	73.00 ± 4.94
MI_theta_cor	69.31 ± 5.13

Note: cor: feature of Pearson correlation; MI: motor imagery.

**Table 2:** Classification accuracy of different number of features.

Number of features	Mean classification accuracy (%)
6	87.81 ± 4.06
8	88.92 ± 3.81
10	92.69 ± 1.37
12	89.94 ± 2.31
14	91.06 ± 1.56

**Table 3:** Selected features from EEG overall energy correlation (left) and beta correlation (right).

Feature number of overall energy correlation	Corresponding lead	*p* value	Feature number of beta correlation	Corresponding lead	*p* value
137	F7-T3	5.8*e* − 34	167	T6-Fz	1.6*e* − 21
123	O1-T6	1.4*e* − 33	123	O1-T6	6.7*e* − 19
155	T3-Fz	4.1*e* − 25	147	F8-T6	2.1*e* − 18
106	P4-O1	2.9*e* − 12	140	F7-T6	9.2*e* − 19
74	C3-T3	1.2*e* − 17	137	F7-T3	3.6*e* − 15
167	T6-Fz	0.0074	114	P4-Cz	0.16
154	T3-Cz	2.0*e* − 18	155	T3-Fz	9.5*e* − 12
126	O1-Pz	9.0*e* − 05	132	O2-T6	1.7*e* − 13
124	O1-Cz	5.1*e* − 14	154	T3-Cz	3.6*e* − 09
162	T5-T6	3.1*e* − 14	116	P4-Pz	4.3*e* − 05

**Table 4:** Mean value of EEG overall energy correlation (left) and beta correlation (right).

Lead of overall energy correlation	Training group	Baseline group	Lead of beta correlation	Training group	Baseline group
F7-T3	0.45	0.67	T6-Fz	0.34	0.28
O1-T6	0.54	0.37	O1-T6	0.58	0.35
T3-Fz	0.34	0.53	F8-T6	0.31	0.29
P4-O1	0.54	0.42	F7-T6	0.43	0.25
C3-T3	0.47	0.64	F7-T3	0.50	0.68
T6-Fz	0.20	0.24	T3-Fz	0.40	0.57
T3-Cz	0.36	0.51	O2-T6	0.88	0.77
O1-Pz	0.56	0.49	T3-Cz	0.43	0.57
O1-Cz	0.33	0.21	P4-Pz	0.85	0.78
T5-T6	0.45	0.32			
